# Results of a Study Comparing Glycated Albumin to Other Glycemic Indices

**DOI:** 10.1210/clinem/dgz087

**Published:** 2019-10-25

**Authors:** Cyrus V Desouza, Richard G Holcomb, Julio Rosenstock, Juan P Frias, Stanley H Hsia, Eric J Klein, Rong Zhou, Takuji Kohzuma, Vivian A Fonseca

**Affiliations:** 1 University of Nebraska Medical Center, Omaha, Nebraska; 2 Quintiles Consulting, Inc., Rockville, Maryland; 3 Dallas Diabetes Research Center at Medical City, Dallas, Texas; 4 National Research Institute, Los Angeles, California; 5 Capital Medical Center, Olympia, Washington; 6 Medpace, Inc., Cincinnati, Ohio; 7 Asahi Kasei Pharma, Tokyo, Japan; 8 Tulane University Health Sciences Center, New Orleans, Louisiana 70112

## Abstract

**Context:**

Intermediate-term glycemic control metrics fulfill a need for measures beyond hemoglobin A1C.

**Objective:**

Compare glycated albumin (GA), a 14-day blood glucose measure, with other glycemic indices.

**Design:**

24-week prospective study of assay performance.

**Setting:**

8 US clinics.

**Participants:**

Subjects with type 1 (n = 73) and type 2 diabetes (n = 77) undergoing changes to improve glycemic control (n = 98) or with stable diabetes therapy (n = 52).

**Interventions:**

GA, fructosamine, and A1C measured at prespecified intervals. Mean blood glucose (MBG) calculated using weekly self-monitored blood glucose profiles.

**Main Outcome Measures:**

Primary: Pearson correlation between GA and fructosamine. Secondary: magnitude (Spearman correlation) and direction (Kendall correlation) of change of glycemic indices in the first 3 months after a change in diabetes management.

**Results:**

GA was more concordant (60.8%) with changes in MBG than fructosamine (55.5%) or A1C (45.5%). Across all subjects and visits, the GA Pearson correlation with fructosamine was 0.920. Pearson correlations with A1C were 0.655 for GA and 0.515 for fructosamine (*P* < .001) and with MBG were 0.590 and 0.454, respectively (*P* < .001). At the individual subject level, Pearson correlations with both A1C and MBG were higher for GA than for fructosamine in 56% of subjects; only 4% of subjects had higher fructosamine correlations with A1C and MBG. GA had a higher Pearson correlation with A1C and MBG in 82% and 70% of subjects, respectively.

**Conclusions:**

Compared with fructosamine, GA correlates significantly better with both short-term MBG and long-term A1C and may be more useful than fructosamine in clinical situations requiring monitoring of intermediate-term glycemic control (NCT02489773).

Hemoglobin A1C and self-monitoring of blood glucose (SMBG) are well established as complementary gold standard metrics for assessing glycemic control (1). However, there is a wide gap between daily SMBG measurements and the average glycemia over 2 to 3 months measured with A1C. The accuracy of A1C may also be affected by variant hemoglobins, anemia, and other medical conditions affecting erythrocyte survival (2). Continuous glucose monitoring (CGM) data can bridge this gap, but CGM devices are used by only a minority of patients with type 1 diabetes and very few with type 2 diabetes (3, 4). Recent proposals for additional metrics beyond A1C have suggested use of intermediate-term assessments, including glycated albumin (GA) and fructosamine, to complement the information provided by A1C and daily monitoring with SMBG or CGM (5, 6).

Serum albumin has a half-life of approximately 14 days. GA therefore represents an intermediate measure between A1C and SMBG (2). The Lucica^®^ Glycated Albumin–L test is an enzymatic assay in which endogenous glycated amino acids and peroxide are eliminated by a ketoamine oxidase and peroxidase reaction. The GA is then hydrolyzed to amino acids or peptides by an albumin-specific proteinase and measured quantitatively. The GA value presented is a ratio (mmol/mol) of total albumin concentration measured in the same serum sample, thereby minimizing effects of variations among individuals and in albumin concentrations (7, 8).

A small-scale pilot study involving 30 subjects assessed the performance of GA measured with the Lucica GA-L by comparing it with other glycemic control metrics (including A1C, fructosamine, and SMBG data) during intensification of antihyperglycemic therapy in patients with type 1 or 2 diabetes for 12 weeks and validated the testing protocol (9). This study evaluated the performance of GA in a larger population over a longer period of time. GA was compared with traditional short-, intermediate-, and long-term markers of glycemic control in 2 groups of patients with type 1 or type 2 diabetes whose treatment was either (1) likely to be intensified or (2) likely to remain stable over 6 months.

## Materials and Methods

### Study design

This was a prospective, multicenter, comparative study of assay performance in a clinical setting conducted at 8 sites (NCT02489773). Subjects with type 1 or type 2 diabetes were recruited in equal numbers and divided into Group 1, comprising at least 90 patients with A1C ≥7.5% who were prescribed a change in diabetes management, and Group 2, including at least 40 patients with A1C <7.5% on a stable therapeutic regimen with no changes planned for the duration of the study. The institutional review boards at each study center approved the protocol and consent form. All patients provided written informed consent.

Over 24 weeks, subjects’ blood was drawn at prespecified intervals and tested in a central laboratory for GA, fasting blood glucose (FBG), fructosamine (uncorrected for albumin), and A1C. SMBG data were collected at each visit; study subjects and investigators followed conventional practices used at each study site for routine SMBG. CGM data for a subset of subjects (at least 30 patients) assigned to masked CGM were also collected to verify the accuracy of mean blood glucose (MBG) readings collected using SMBG. GA results were blinded from study participants and not used in the diagnosis or management of subjects with diabetes.

### Subjects

Eligible participants were men or women ≥18 years of age with either type 1 or type 2 diabetes (the minimum age was 19 years at the site in Nebraska). Group 1 subjects included those with A1C between 7.5% and 12.0% at screening for whom the study investigator was already planning to institute or was in the process of instituting therapy to improve glycemic control with oral agents, insulin, or noninsulin injectable antihyperglycemic medications. Subjects enrolled in Group 2 had an A1C <7.5% at screening and were on a stable diabetes treatment regimen, with no changes in the 3 months prior to screening and no plans to change the regimen during the 24-week study. Patients were permitted to adjust insulin doses according to daily needs.

Patients were excluded if the investigator judged them to have any clinically significant disease that would interfere with study evaluations or ongoing treatment for other medical conditions, including chronic kidney or end-stage renal disease, liver cirrhosis, uncontrolled thyroid disease, anemia, a known hemoglobinopathy, blood transfusion in the last 6 months, or any other acute or chronic condition that might significantly influence albumin or glucose metabolism. Routine iron deficiencies were not exclusion criteria, and investigators used their clinical judgment to decide whether to enroll patients with these abnormalities.

### Endpoints

The primary endpoint was the mean Pearson correlation coefficient across all study visits during the 6-month study period by subject; equivalent performance of the GA and fructosamine assays was demonstrated by a mean Pearson correlation >0.8. Because predicate fructosamine assays cleared by the FDA were uncorrected for albumin, uncorrected fructosamine values were used in the primary analyses. Two hierarchically tested secondary endpoints evaluated the magnitude and direction of change of glycemic indices in the first 3 months after a change in diabetes management in Group 1. The first secondary endpoint compared the Spearman rank correlation coefficient between the GA assay and MBG, as determined by daily SMBG levels, with the correlation between conventional A1C and MBG; the endpoint was met if the Spearman coefficient for GA was larger than that for A1C. The next secondary endpoint assessed the concordance of changes (same direction of increases or decreases across all pairs of observations) in GA vs MBG and in A1C vs MBG using the Kendall tau rank correlation coefficient; the endpoint was met if the coefficient for GA was larger than the A1C coefficient.

Additional secondary endpoints included the primary endpoint stratified by Groups 1 and 2 and by diabetes type; Pearson correlations from linear regressions of GA with other glycemic measures, including FBG, MBG, and A1C values; comparison of SMBG- vs CGM-derived MBG; correlations between changes from baseline for GA, fructosamine, FBG, and A1C; and the relationship between early changes in GA and long-term changes in A1C.

### Assessments

Fasting blood samples drawn at Weeks 0 (screening), 1 (study start), 2, 3, 4, 6, 8, 12, 16, 20, and 24 were tested for GA, fructosamine, FBG, and A1C. SMBG data were also collected at each visit for the period since the last visit. Visits were at 1-week intervals during the first month to capture more rapid changes expected with initiation of therapy. Intervals were lengthened to 2 weeks during the second month and to 4 weeks during months 3 to 6. Participants conducted routine SMBG, according to their normal monitoring schedule, using a blood glucose meter with memory capabilities (OneTouch^®^ Ultra^®^ 2 Blood Glucose Meter [LifeScan, Inc., Milpitas, CA]) supplied by the investigators. Participants were also instructed to use their SMBG device 7 times at least 1 day per week, collecting 3 preprandial, 3 postprandial, and 1 bedtime measurement. During the weeks when a study visit was scheduled, patients were asked to conduct the 7-point SMBG measurement the day before the scheduled study visit.

A subset of participants also used a masked CGM device (Dexcom G4™ PLATINUM CGM System [Dexcom Inc., San Diego]) beginning at enrollment (Week 1) and continuing for the full 24-week study period. Subjects placed the sensors themselves if they were comfortable doing so. 

Participants attended each study visit in the fasting state, and the following were collected: vital signs (blood pressure, heart rate); whole blood, serum, and plasma for assays of FBG, fructosamine, GA, and A1C; and SMBG data. Blood was allowed to clot at room temperature for 45 minutes, centrifuged at 1800*g* for 15 minutes, then frozen at ≤70°C prior to shipment to the central laboratory (Medpace Reference Laboratories, Cincinnati, OH) where all samples were analyzed. Plasma was refrigerated at 2 to 8°C before shipment to the laboratory. GA and fructosamine tests were performed on serum samples. A1C and glucose tests were performed on whole blood (EDTA-2K) and plasma, respectively. The GA value was measured using a Roche/Hitachi Modular P instrument and determined using the Lucica Glycated Albumin-L assay (Asahi Kasei Pharma Corporation, Tokyo, Japan); it was reported in mmol/mol and converted to percentage values using the formula GA (%) = 0.05652 × GA (mmol/mol) – 0.4217 (10). This GA assay was traceable to reference material certified by the Committee on Diabetes Mellitus Indices of the Japan Society of Clinical Chemistry (11). A1C was determined using the G7 and G8 high-performance liquid chromatography analyzers (Tosoh Bioscience, Inc., San Francisco, CA), which are National Glycohemoglobin Standardization Program (NGSP)-certified methods. FBG was analyzed by photometry using reagents OSR6221 with Beckman Coulter AU2700/5800 (Beckman Coulter Diagnostics, Brea, CA). Fructosamine was determined with the Randox Fructosamine reagent kit using Randox Daytona (Randox, UK), and the serum albumin (for fructosamine correction) was determined with the Beckman Coulter AU series chemistry analyzer using the Beckman Coulter reagent kit. The coefficients of variation for these reagents and instruments were <2%.

### Statistical analysis

Descriptive statistics were used to summarize all results in this study. Continuous variables were summarized by sample size, mean, standard deviation, median, minimum, and maximum. Categorical variables were summarized by number and percentage. To enable the comparison of changes from baseline in indices with different units of measure over time, differences between values at baseline and at each visit were converted to percentage changes from baseline. 

MBG was estimated as the average of readings taken during successive 7-day intervals between study visits, from home monitoring measurements performed by subjects and uploaded from their SMBG device. The 7-day MBG based on SMBG was calculated with the MBG of all SMBG values in a 7-day interval. The interval could include ≤7 points in a day; all values in the interval between visits were included in the calculation of MBG. MBG was estimated similarly from CGM data uploaded from participants’ Dexcom devices and was also estimated as the average of readings taken during successive 7-day intervals between study visits. 

The primary endpoint was statistically tested by comparing the mean Pearson correlation coefficient of GA with fructosamine from individual study subjects to a prespecified performance goal (≥0.8), which was proposed as a minimum threshold value to demonstrate evidence of the clinical equivalence of GA with fructosamine. The method for estimating the mean Pearson correlation using the within-subject correlations was included in the original Investigational Device Exemption (IDE) protocol; however, it was found to be statistically invalid. A revised, statistically valid and unbiased method of estimating the mean Pearson correlation, as well as estimating the Spearman and Kendall correlation coefficients, using the randomized resampling method of Lorenz et al. (12), was implemented.

A 2-sided 95% confidence interval (CI) of the Pearson correlation of GA and fructosamine was constructed based on its mean and standard error, assuming the individual corrections followed a normal distribution. It was concluded that the Pearson correlation of GA and fructosamine was at least 0.8 if the lower bound of the 95% CI was greater than 0.80. 

The secondary endpoints comparing the magnitude and direction of changes in GA, MBG, and A1C were planned to be hierarchically tested if the primary endpoint was met. The difference in Spearman and Kendall correlations between GA and MBG vs A1C and MBG in the first 3-month period in Group 1 was tested using the following Wald test statistic: test statistic = (C_GA_ – C_A1C_)/SE (C_GA_ – C_A1C_), in which (C_GA_ – C_A1C_) was the observed difference between C_GA_ and C_A1C_ and SE (C_GA_ – C_A1C_) was the corresponding standard error, which was estimated using the random resampling method of Lorenz et al. (12). A 1-sided *P* value of ≤.025 was considered evidence of statistical significance. 

Given an expected mean Pearson correlation of 0.85, a corresponding standard deviation of 0.16 determined from the pilot study, and a performance goal of 0.80, a sample size of 110 evaluable patients was assumed to be required to achieve 90% power based on a 1-sided, 1-sample Student’s t-test at the type I error level 0.025.

The predictive relationship between changes in GA over Weeks 1 to 4 and long-term changes in A1C (Week 12) was assessed using a logistic regression analysis in which changes in A1C at Week 12 were categorized into a binary variable (<0.5%, ≥0.5%) to be the outcome with a continuous variable (changes in GA at Weeks 1–4) as a covariate. MBG and CGM results were compared using paired differences and Bland Altman plots.

## Results

Out of 165 subjects screened, 150 were enrolled and 141 completed the study. Five subjects were lost to follow-up; others withdrew for personal or investigator-related reasons. None withdrew due to an adverse event. A total of 149 subjects met minimum follow-up requirements to be included in the analysis of study endpoints.

Among enrolled subjects, 98 were assigned to Group 1 (type 1 diabetes, n = 47; type 2 diabetes, n = 51) and 52 to Group 2 (type 1 diabetes, n = 26; type 2 diabetes, n = 26). Slightly more than half of subjects were female; the majority were non-Hispanic whites with a mean age of 51 years (Table 1), although 11% of the population comprised African Americans and 19% were Hispanic. Insulin was used by 69% of the population, and 48% used a noninsulin antihyperglycemic agent. Metformin with or without other agents was used by 46% of subjects. At the start of the evaluation period, 95% of subjects in Group 1 had an A1C ≥7.5% and all subjects in Group 2 had A1C values <7.5%. During the study, the treatment regimens of subjects in Group 1 were adjusted at the investigator’s discretion.

**Table 1.** Subject demographics at baseline.^*a*^

	Group 1 (n = 98)	Group 2 (n = 52)	Overall (n = 150)
Mean age, years (SD)	50.6 (15.61)	50.3 (15.81)	50.5 (15.63)
Female, n (%)	53 (54.1)	27 (51.9)	80 (53.3)
Race			
White, n (%)	80 (81.6)	46 (88.5)	126 (84.0)
Black, n (%)	13 (13.3)	4 (7.7)	17 (11.3)
Asian, n (%)	5 (5.1)	2 (3.8)	7 (4.7)
Ethnicity			
Hispanic	22 (22.4)	7 (13.5)	29 (19.3)
Diabetes type			
Type 1	47 (48.0)	26 (50.0)	73 (48.7)
Type 2	51 (52.0)	26 (50.0)	77 (51.3)
Mean weight, kg (SD)	89.7 (21.90)	90.5 (24.91)	90.0 (22.91)
Mean BMI, kg/m^2^ (SD)	31.5 (6.62)	31.0 (6.77)	31.3 (6.65)
Mean serum albumin, g/L (SD)	45.3 (3.2)	46.2 (3.0)	45.6 (3.1)
Antihyperglycemic use			
Insulin,^*b*^ n (%)	72 (73.5)	31 (59.6)	103 (68.7)
Oral and/or noninsulin injectable agents, n (%)	49 (50.0)	23 (44.2)	72 (48.0)
Glycemic indices			
Mean A1C, % (SD)	8.7 (0.99)	6.6 (0.48)	8.0 (1.29)
mmol/mol (SD)	72 (10.8)	49 (5.2)	64 (14.1)
Mean FBG, mmol/L (SD)	9.91 (3.36)	7.89 (2.79)	9.21 (3.31)
mg/dL (SD)	178.6 (60.56)	142.2 (50.24)	166.0 (59.60)
Median MBG, mmol/L^*c*^ (min, max)	9.6 (6.1, 19.6)	7.6 (5.5, 10.7)	8.8 (5.6, 19.6)
mg/dL (min, max)	172.8 (110.0, 352.6)	136.2 (98.5,191.9)	157.6 (98.5, 352.6)
Mean fructosamine, µmol/L (SD)	459.6 (109.42)	343.5 (73.92)	419.3 (112.86)
Mean GA, mmol/mol (SD)	389.7 (77.79)	285.8 (52.05)	353.7 (85.61)
Mean GA, % (SD)^*d*^	21.6 (3.97)	15.7 (2.52)	19.6 (4.42)

^*a*^Determined at screening visit (Visit 1, prior to Week 0) unless otherwise noted.

^*b*^With or without other antihyperglycemic agents.

^*c*^Median baseline determined at Week 0 (Visit 2).

^*d*^GA (%) = 0.05652 × GA (mmol/mol) – 0.4217 (10).

Abbreviations: BMI, body mass index; FBG, fasting blood glucose; GA, glycated albumin; max, maximum; MBG, mean blood glucose; min, minimum; SD, standard deviation.

### Primary endpoint: Pearson correlation of GA with fructosamine

In the primary endpoint analysis, the within-subject correlation between GA and fructosamine was 0.643. The estimated mean Pearson correlation using the resampling method of Lorenz et al. (12) confirmed the strong correlation between GA and fructosamine (0.9198 ± 0.0135) and exceeded the prespecified performance goal of 0.80 (*P* < .001). In 10 000 resampling trials of study data, the sampled Pearson correlations ranged from a minimum of 0.8628 to 0.9548. When compared across all study patients, GA and fructosamine corrected for albumin were also well correlated (Pearson correlation = 0.9422; *r*^2^ = 0.8878), and the correlation was significantly greater than the performance goal of 0.8 (*P* < .0001).

**Figure 1.**
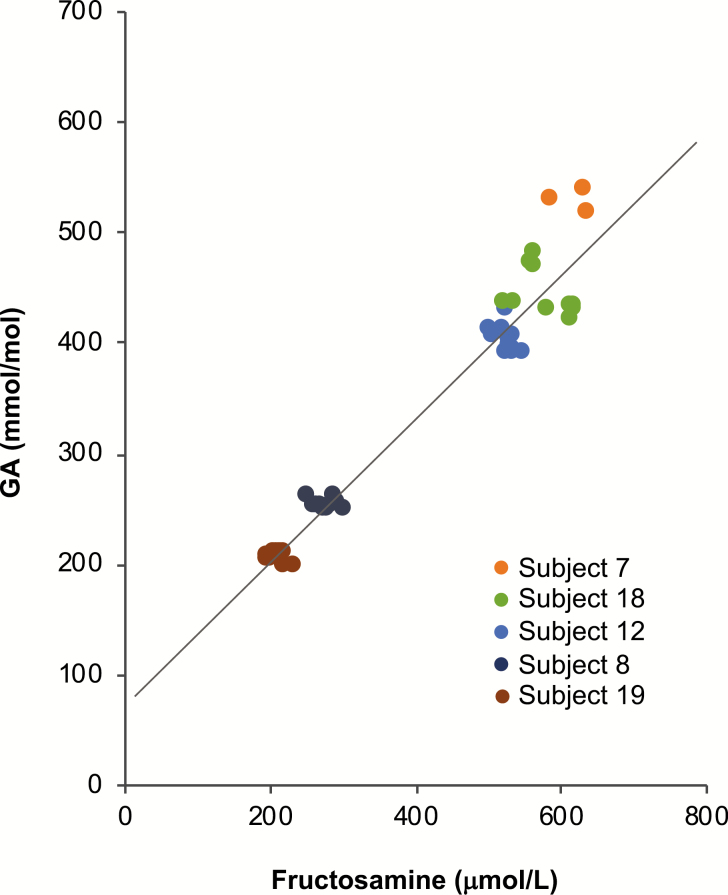
Study subjects with lowest within-in subject Pearson correlations (overall, r = 0.973). FRA = fructosamine. GA = glycated albumin. The line represents the fit for the total population.

The statistical rationale for using the resampling method of Lorenz et al. (12) rather than an analysis based on within-subject correlations is illustrated in Fig. 1, which displays the GA and fructosamine results for the 5 subjects from the population of 149 evaluable subjects with the lowest observed within-subject Pearson correlations. Although the individual within-subject correlations were all negative (range −0.527 to −0.189), the overall Pearson correlation for the group was 0.973. There was high reproducibility between GA and fructosamine paired values for each subject, but the narrow range of reading values made the linear Pearson correlation coefficients within subjects a poor measure of agreement.

The consistency of the Pearson correlations in study groups was also examined using the same resampling method. There was no evidence of major differences in Pearson correlations between subgroups (Table 2). The relative difference in estimated mean Pearson correlations between the subgroups examined (diabetes type, group assignment, gender, race, and age) ranged from approximately 2% to 5%. All estimated mean Pearson correlations exceeded 0.90 for all comparisons except type 1 (0.8804), and the minimum correlation estimates seen in 10 000 resamples of study data exceeded 0.80 for all comparisons except men (minimum, 0.7736) and subjects less than or equal to the median age of 54 years (minimum, 0.7954).

**Table 2.** Pearson correlations of GA with fructosamine in subgroups (10 000 resamples in 149 subjects).

Subgroup	n	Mean	Median	Standard deviation	Minimum	Maximum
Diabetes type						
Type 1	72	0.8804	0.8813	0.0185	0.8055	0.9354
Type 2	77	0.9251	0.9304	0.0247	0.8027	0.9718
Group						
Group 1	97	0.9030	0.9057	0.0198	0.8173	0.9560
Group 2	52	0.9492	0.9503	0.0107	0.8913	0.9787
Gender						
Male	69	0.9117	0.9167	0.0278	0.7736	0.9713
Female	80	0.9289	0.9294	0.0104	0.8833	0.9623
Race						
White	125	0.9169	0.9193	0.0158	0.8494	0.9565
Non-white	24	0.9402	0.9418	0.0171	0.8642	0.9860
Median age						
≤54 years	74	0.9055	0.9100	0.0234	0.7954	0.9619
>54 years	75	0.9316	0.9324	0.0133	0.8774	0.9688

### Secondary endpoints

In the first secondary endpoint analysis of the magnitude of the changes occurring in Group 1 during the first 3 months of the study, Spearman correlations were 0.481 between GA and MBG and 0.233 between A1C and MBG, with a statistically significant difference of 0.249 (95% CI 0.130–0.367; *P* < .0001).

In the next secondary endpoint analysis of the direction of change analysis, Kendall correlations were greater between GA and MBG (0.341) than A1C and MBG (0.160), with a significant difference of 0.181 (95% CI 0.096–0.265; *P* <.0001).

A logistic regression analysis comparing decreases in GA over Weeks 1 to 4 and a ≥0.5% decrease in A1C at Week 12 (in Group 1) showed increasingly strong associations with GA measured at Week 4. The odds ratio of change in GA at Week 4 from baseline was 3.80 (95% CI 1.324–10.904). 

### Other analyses

A total of 35 individuals wore blinded CGM devices for the 24-week study period, and at each visit, MBG obtained by CGM was compared with MBG determined by daily and 7-day SMBG. Out of a total of 284 comparisons of CGM and daily SMBG over 12 visits, the estimated difference was 0.13 mmol/L (2.4 mg/dL; 95% CI –0.07 to 0.34 mmol/L [–1.2 to 6.0 mg/dL]). Out of 313 comparisons of CGM and 7-day SMBG, the estimated difference was 0.16 mmol/L (2.9 mg/dL; 95% CI 0.04–0.28 mmol/L [0.7–5.1 mg/dL]). In this study MBG and CGM were observed to be of equal value in summarizing subject blood glucose levels between study visits.

### Relative performance of GA and fructosamine

**Figure 2.**
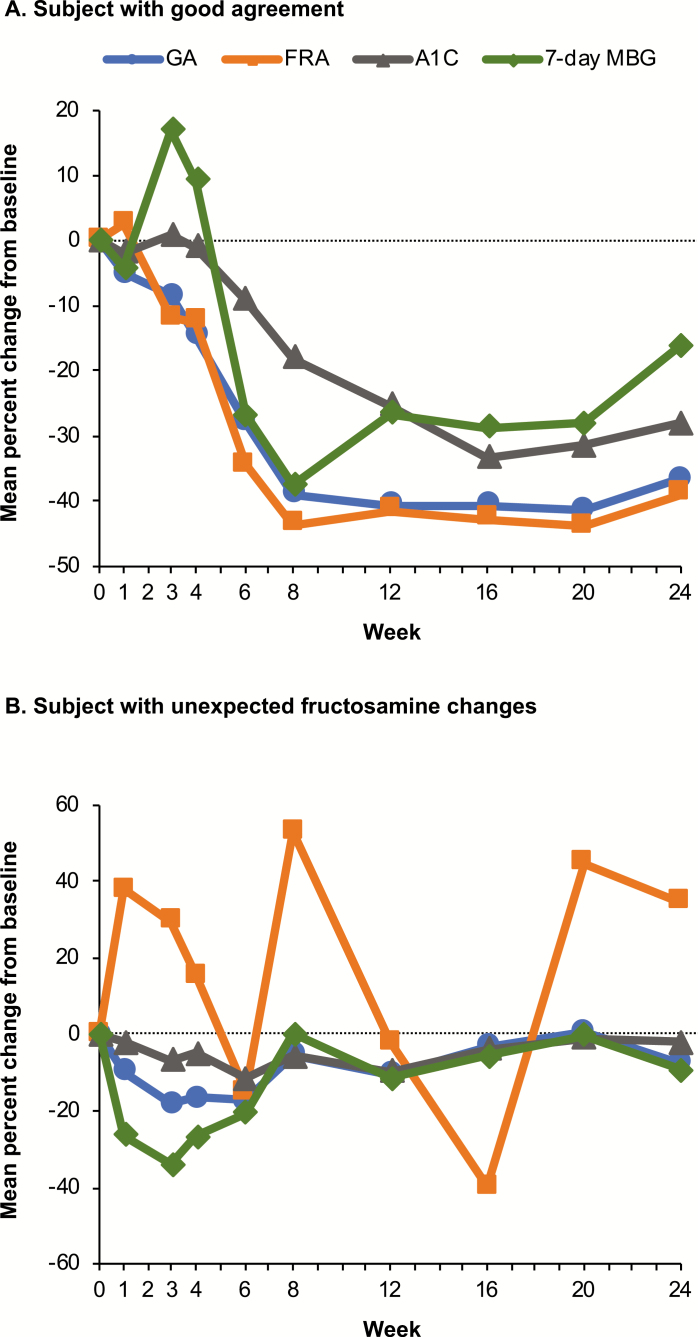
Single subject data for all indices over time. (A) Example of a subject with good agreement between percent changes in all indices. (B) Example of a subject with unexpected changes in fructosamine results. FRA, fructosamine; GA, glycated albumin; MBG, mean blood glucose.

Although there was a high level of agreement between GA and fructosamine across study subjects as measured by the Pearson (0.9198), Spearman (0.9491), and Kendall correlations (0.7639), GA consistently had higher correlations with A1C and MBG than fructosamine (Table 3). Within subjects, the correlations for GA with A1C and MBG were significantly greater than those observed for fructosamine (0.585 vs 0.395 for A1C for GA and fructosamine, respectively [*P* < .001], and 0.548 vs 0.413 for MBG for GA and fructosamine, respectively [*P* < .001]). Although there was generally a high level of agreement in changes over time in all 4 glycemic measures (GA, fructosamine, A1C, and MBG) (Fig. 2A), fructosamine values sometimes departed from expectation based on the other 3 indices (Fig. 2B). Departures for fructosamine versus the other three indices were observed to occur in approximately 9% of subjects (13/149). Unexpected variability in GA changes was not observed.

**Table 3.** **Summary of correlation analyses across study visits by resampling method of Lorenz et al.** (12).

Type	Mean	Median	Standard deviation	Minimum	Maximum
**Pearson correlations**					
GA with FRA	0.9198	0.9217	0.0135	0.8628	0.9548
GA with A1C	0.6551	0.6555	0.0269	0.5431	0.7451
FRA with A1C	0.5153	0.5164	0.0385	0.3509	0.6402
GA with MBG	0.5902	0.5902	0.0345	0.4614	0.7088
FRA with MBG	0.4540	0.4565	0.0508	0.2317	0.6242
A1C with MBG	0.6897	0.6908	0.0374	0.5235	0.8163
**Spearman correlations**					
GA with FRA	0.9491	0.9531	0.0156	0.8842	0.9763
GA with A1C	0.7193	0.7180	0.0442	0.5758	0.7991
FRA with A1C	0.6100	0.6216	0.0574	0.4622	0.7143
GA with MBG	0.7452	0.7547	0.0570	0.5745	0.8289
FRA with MBG	0.6735	0.6869	0.0782	0.4055	0.7889
A1C with MBG	0.7183	0.7208	0.0466	0.5712	0.8088
**Kendall correlations**					
GA with FRA	0.7639	0.7644	0.0164	0.7283	0.8109
GA with A1C	0.4536	0.4536	0.0219	0.4066	0.5135
FRA with A1C	0.3437	0.3475	0.0274	0.2642	0.3961
GA with MBG	0.4144	0.4159	0.0271	0.3470	0.4634
FRA with MBG	0.3310	0.3367	0.0342	0.2384	0.4018
A1C with MBG	0.5043	0.5051	0.0319	0.4360	0.5862

Abbreviations: GA, glycated albumin; FRA, fructosamine; MBG, mean blood glucose.

**Figure 3.**
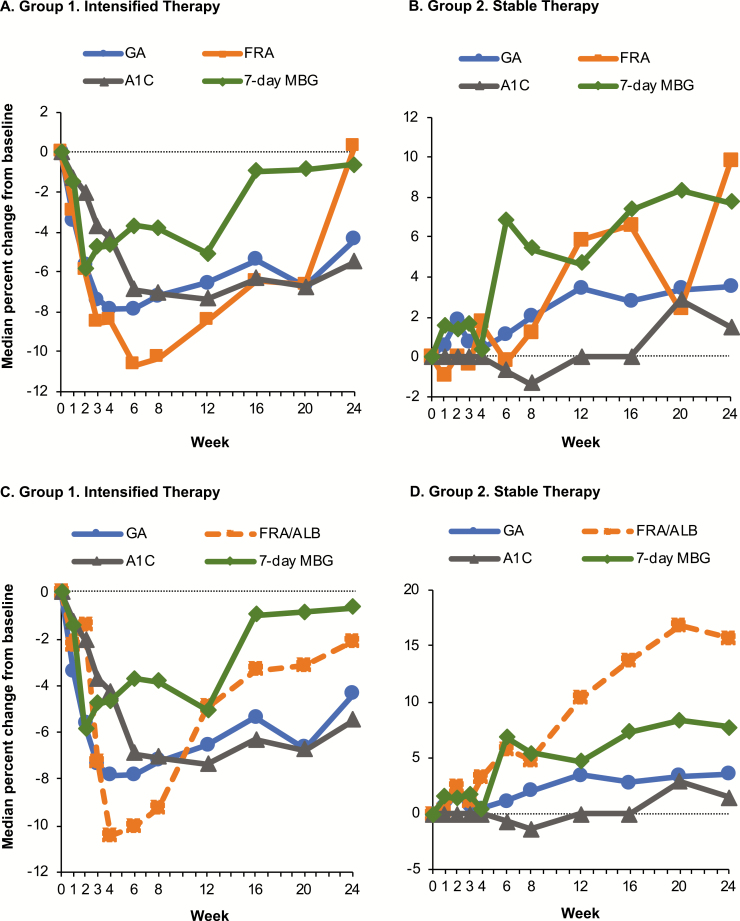
Median percent change in glycated albumin (GA), fructosamine (FRA), mean blood glucose (MBG), and A1C. Percent changes are used so that all indices can be shown on the same scale. (A) Group 1 with uncorrected fructosamine. (B) Group 2 with uncorrected fructosamine. (C) Group 1 with fructosamine corrected for albumin (FRA/ALB). (D) Group 2 with fructosamine corrected for albumin.

Figure 3 shows median percentage changes of all indices for both groups. In Group 1 (Fig. 3A), after an initial decrease in all groups, MBG began to increase gradually at Week 2 and more precipitously at Week 12. GA began increasing after Week 4, reflecting the rise in MBG sooner than either fructosamine (which reached its nadir at Week 6) or A1C, which reached its nadir value at Week 12. In Group 2 (Fig. 3B), MBG began rising after Week 4. This change was reflected sooner and more consistently by GA than either fructosamine or A1C. Changes in GA between study visits were concordant (increased or decreased in the same direction) with MBG changes 60.8% of the time, with fructosamine changes 55.5% of the time, and with A1C 45.5% of the time. Changes in fructosamine corrected for albumin appeared to show a more exaggerated decrease than any other glucose measure in the intensified therapy group. Similarly, in the stable therapy group, fructosamine corrected for albumin reflected a larger increase in blood glucose than any other measure (Fig. 3C and 3D).

Overall, the Pearson correlation between GA and A1C was higher than the correlation between fructosamine and A1C in 122 (81.9%) subjects, and the correlation between GA and MBG was higher than that between fructosamine and MBG in 105 (70.5%) subjects. GA had a higher correlation than fructosamine with both A1C and MBG in 84 (56.4%) subjects, whereas fructosamine had higher correlations with A1C and MBG in 4.0% of subjects.

## Discussion

This 24-week study confirmed results of a small-scale, 12-week pilot study (9), showing that GA and fructosamine are well correlated and can both be used to assess glycemic control in a shorter time frame than A1C. The study met both its primary and secondary endpoints and demonstrates that GA may represent an improved clinical application for intermediate-term measurement of blood glucose versus fructosamine. Changes in GA reflected short-term fluctuations in MBG and CGM and also predicted long-term changes in A1C more consistently than fructosamine in subjects whose treatment regimens were adjusted to improve glycemic control (Group 1) and in those with stable therapy (Group 2). Diabetes type (1 or 2) did not affect assay results.

Why GA performed better than fructosamine in this study requires further investigation. Besides albumin, fructosamine levels may fluctuate in response to other serum proteins (5, 13, 14). This factor may have played a role in the unexpected variation in fructosamine seen in this study. Because GA is specific to albumin, it may be less influenced by variations in other molecules (5). Although these assays were performed in the same laboratory for this research study in a standardized way, in current, real-world practice, fructosamine assays lack standardization (14). The GA assay tested in this study is traceable to reference material that has the potential for standardization if other GA methods become available.

Although A1C remains the gold standard glycemic control measure, its limitations—such as the inability to capture short-term variations in glycemic control or hypoglycemic or hyperglycemic events—have prompted increased consideration of complementary assessments. In a recent consensus document on outcome measures in type 1 diabetes, an international panel of experts recommended CGM data and patient-reported outcomes be considered alongside A1C when evaluating patient health (15). While CGM is increasingly used by patients with type 1 diabetes (3, 16), the technology is rarely reimbursed for patients with type 2 diabetes and thus infrequently used by this population (4).

In this study, GA was better correlated and had better concordance (ie, direction of change up or down) with MBG than other glycemic indices. The lowest observed concordance of 45.5% for A1C and MBG is consistent with the lifespan of red blood cells versus the time response for GA and fructosamine. The ~5% difference between concordance percentages for GA and MBG (60.8%) and fructosamine and MBG (55.5%) reflects the high correlation between GA and fructosamine shown by other summary measures reported herein. GA consistently had equal or better agreement with MBG than fructosamine, an important prerequisite if use of GA is being considered as an alternative to fructosamine. GA could serve as a complementary measure to determine sooner if a treatment strategy is not working. For example, it could be useful during insulin titration, especially for patients who are unable or unwilling to perform regular SMBG or wear a CGM device (17). Delays in treatment intensification can expose patients to extended periods of glycation, increasing their risk of diabetes complications (18–20). GA may have similar utility to A1C in the prediction of complications risk, as both prospective and observational studies have established the association between GA elevations and increased risk of microvascular and macrovascular complications and mortality (21–27). GA may also serve as a substitute in patients with hemoglobinopathies and other conditions in which A1C measurement is unreliable (5, 28). Moreover, in African American and Hispanic individuals, the relationship between A1C and estimated average glucose (eAG) may differ from the pattern observed in non-Hispanic white patients (29–31). The present study was designed to address some of these gaps. It is the first prospective trial of GA and the largest conducted to date, and it was designed to compare GA with other glycemic indices not only across all subjects but also at the individual level. The study population also was recruited to reflect the demographic make-up of US patients with diabetes, including African Americans and Hispanics.

GA has some limitations as a glycemic measure. First, the prognostic cutoffs for GA have not yet been established (1, 5). Glycated proteins, including albumin, are elevated relative to blood glucose levels in patients with liver cirrhosis but decreased in patients with liver failure and nonalcoholic fatty liver disease (32–34). The results of this study are limited to the study population, which excluded patients with liver cirrhosis and nephrosis. However, there is no known problem in patients with mild fatty infiltration of the liver, which is common in type 2 diabetes. No prior screening with ultrasound was required for this study, and as such most patients in routine clinical practice outside centers treating advanced liver disease could have been included. GA levels may also be reduced in patients with nephrosis, severe hypertriglyceridemia, and any other condition influenced by albumin catabolism (35–39). However, triglyceride elevations ≤392 mmol/L (≤1516 mg/dL) do not interfere with the GA assay used in this study (40).

In summary, GA was a strong indicator of overall glucose control in people with diabetes, reflecting intermediate-term (2–4 weeks) changes in average glycemia in a manner comparable to currently available measures. The assay accurately reflects glycemic control in both type 1 and type 2 diabetes, whether their antihyperglycemic regimens are stable or being changed to improve control. GA levels also predict future A1C, which may be helpful to clinicians wishing to evaluate early treatment responses or predict deteriorations in glycemic control.

## Abbreviations

CGM: continuous glucose monitoring
CI: confidence interval
eAG: estimated average glucose
FBG: fasting blood glucose
GA: glycated albumin
MBG: mean blood glucose
SE: standard error
SMBG: self-monitoring of blood glucose.

## Additional Information

***Disclosure Summary:*** C.V.D. has received research grants and consulting fees from NovoNordisk and research grants from KOWA, THERACOS, the National Institutes of Health (NIH), and Sanofi. R.G.H. received consulting fees from Asahi Kasei Pharma Corporation. J.R. has served on scientific advisory boards and received honoraria or consulting fees from Eli Lilly, Sanofi, Novo Nordisk, Janssen, AstraZeneca, Boehringer Ingelheim, and Intarcia and has received grants/research support from Merck, Pfizer, Sanofi, Novo Nordisk, Bristol-Myers Squibb, Eli Lilly, GlaxoSmithKline, Genentech, Janssen, Lexicon, Boehringer Ingelheim, and Intarcia. J.P.F. received research support from Asahi Kasei Pharma Corporation through his institution. S.H.H. received research support from Asahi Kasei Pharma Corporation through his institution. E.J.K. has no interests to disclose. R.Z. is employed by Medpace, which was paid by Asahi Kasei Pharma Corporation to perform the statistical analysis. T.K. is an employee of Asahi Kasei Pharma Corporation. V.A.F. has received research grants from Bayer and Boehringer Ingelheim through his institution and directly received honoraria for consulting and lectures from Takeda, Novo Nordisk, Sanofi, Eli Lilly and Company, Astra Zeneca, Intarcia, and Asahi Kasei Pharma Corporation.

***Data Availability:*** All data generated or analyzed during this study are included in this published article or in the data repositories listed in References.
